# Inhibitory Effects of Resveratrol Analogs on Mushroom Tyrosinase Activity

**DOI:** 10.3390/molecules171011816

**Published:** 2012-10-09

**Authors:** Danielle Cristina Zimmermann Franco, Gustavo Senra Gonçalves de Carvalho, Paula Rafaela Rocha, Raquel da Silva Teixeira, Adilson David da Silva, Nádia Rezende Barbosa Raposo

**Affiliations:** 1 NIQUA, Federal University of Juiz de Fora, Juiz de Fora, Minas Gerais 36036-900, Brazil; Email: dannyzimmermann@yahoo.com.br (D.C.Z.F.); rrocha.paula@hotmail.com (P.R.R.); raquelufjf@gmail.com (R.S.T.); 2 Department of Chemistry, I.C.E., Federal University of Juiz de Fora, Campus Universitário, Juiz de Fora, Minas Gerais 36036-900, Brazil; Email: gustavo.carvalho@ice.ufjf.br

**Keywords:** chemical synthesis, mushroom tyrosinase activity, structure-activity, tyrosinase, activity *in vitro*

## Abstract

Skin pigmentation disorders typically involve an overproduction or uneven distribution of melanin, which results in skin spots. Resveratrol can inhibit tyrosinase, the active enzyme in the synthesis of melanin, but it does not inhibit the synthesis of melanin to an extent that enables its use alone as a skin whitening agent in pharmaceutical formulations, so its use as a coadjuvant in treatment of hyperpigmentation is suggested. Six resveratrol analogs were tested for tyrosinase inhibitory activity *in vitro*. Among the analogs tested, compound **D** was the most powerful tyrosinase inhibitor (IC_50_ = 28.66 µg/mL), two times more active than resveratrol (IC_50_ = 57.05 µg/mL), followed by the analogs **A**, **E**, **B**, **F** and **C**, respectively. This demonstrated that the hydroxylation at C4' on the phenolic ring was the molecular modification with most importance for the observed activity.

## 1. Introduction

The appearance of the skin is broadly associated with beauty. Therefore, the desire to have a skin looking healthy and free of spots is the goal of many individuals, especially women [1]. Skin pigmentation disorders consist of an overproduction or uneven distribution of melanin, which results in skin spots that mainly affect mainly the face [2]. Such conditions may arise due to numerous factors, including skin aging, sun exposure [3,4], genetic factors [5], ethnicity [6,7], pregnancy [8], disease [9,10], use of certain medicines [11] and others.

**Figure 1:**
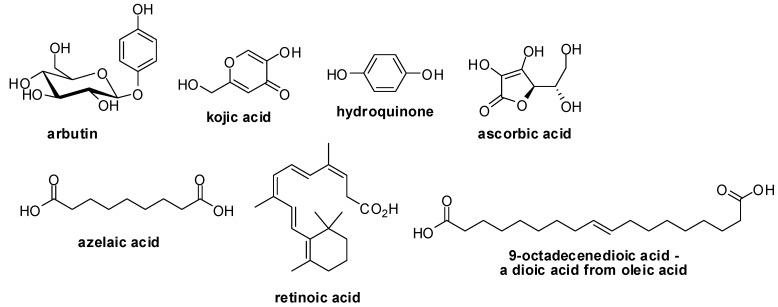
Structures of commercially used depigmenting agents.

The main options for the treatment of skin hyperpigmentation include topical agents, chemical peels, cryotherapy and laser therapy [4]. The majority of the cosmetics utilized in topical treatment of hyperpigmentation contain in their formulation substances such as arbutin, azelaic acid, hydroquinone and kojic acid, with the last two being the main therapeutic options used for depigmenting [12,13]. These substances work by inhibiting the tyrosinase, an enzyme that catalyses the oxidation of tyrosine [12,13]. Others agents, such as dioic acid (derived from oleic acid), ascorbic acid, retinoic acid and soy extracts, act through different mechanisms (Figure 1) [14].

However, the use of hydroquinone and kojic acid are associated with some adverse reactions that may aggravate the appearance of the spots and damage the health of the user. Also, the systemic and collateral effects of these substances have not been fully elucidated [15,16]. For these reasons, the use of cosmetics that contain hydroquinone is prohibited in the European Union and is strictly controlled in the United States by the Food and Drug Administration (FDA) [17]. Instead, dioic acid has been used to treat hyperpigmentation, with good efficacy, but with similar side effects as hydroquinone [18].

The compounds utilized in the treatment of hyperpigmentation usually act as competitive or noncompetitive inhibitors of tyrosinase and thereby prevent the conversion of tyrosine into 3,4-dihydroxyphenylalanine (L-DOPA), and of L-DOPA into dopaquinone, that occur through hydroxylation and oxidation reactions, respectively. Once they block these reaction steps, they also block melanin synthesis [3,13,18,19].

Resveratrol is a polyphenolic phytoalexin that belongs to the stilbenes, which have demonstrated potent antioxidant activity [20,21,22], and numerous pharmacological activities [23,24,25,26], including tyrosinase inhibitory activity. Their hydroxyl derivatives, including oxyresveratrol, also have the same activities and have shown potent inhibitory effects on tyrosinase activity [13,27]. The substances in this drug class act as competitive inhibitors of tyrosinase, in the presence of tyrosine and L-DOPA. After undergoing the enzymatic reaction, its metabolites act as noncompetitive inhibitors of tyrosinase, considering the substrate L-DOPA [13,28]. Furthermore, an investigation using B16-F10 murine melanoma cells showed cellular melanin production was significantly suppressed by resveratrol without any cytotoxicity up to 200 μM [28].

However, some studies have demonstrated that resveratrol does not inhibit the synthesis of melanin to such a degree that enables it to be utilized alone as skin whitening agent in pharmaceutical formulations, and so its use as a coadjuvant in hyperpigmentation treatments is suggested [13,28].

Due to the potential pharmacology presented by resveratrol and its analogues [29], also referred to as stilbene analogs, our research group in a recent effort has been developing the synthesis and biological evaluation of synthetic resveratrol analogs, particularly the aza-stilbenes, bioisosteres [30] of natural stilbenes, with a view to improving the potential of the natural analogues of resveratrol. Such compounds have shown good antitubercular and antioxidant activity [21,31,32], which encouraged us to test the potential of this class of compounds in other areas, such as depigmenting action. Thus, this paper presents a study of the *in vitro* inhibitory activity of six aza-stilbenes or azaresveratrols analogs on tyrosinase activity.

## 2. Results and Discussions

All the analogs presented inhibitory concentration to 50% of tyrosinase (IC_50_) values greater than 50% in screening (qualitative assay). The tyrosinase inhibitory activity results demonstrated that the analyzed azaresveratrol analogs **A**–**F** showed a greater capacity to inhibit tyrosinase more than kojic acid (*p* < 0.05) during the first hour of the qualitative assay. In the second hour of quantitative assay, the analogs **D**, **E** and **F** showed inhibitory ability statistically equal to that of kojic acid (*p* > 0.05) (Table 1).

Analog **D** presented the greatest tyrosinase inhibition potency (IC_50_ = 28.66 μg/mL), followed by analogs **A**, **E**, **B**, **F** and **C**, respectively. Furthermore, it showed a more stable IC_50_ in the quantitative assay compared with kojic acid. The performance of analog **D** may be associated to its known antioxidant capacity, as demonstrated in a previous study conducted by our group [21]. The presence of a hydroxyl group at the *para*-position of the aromatic ring thus appears to be critical to good antioxidant activity, as well as tyrosinase inhibitors. This is an expected result since resveratrol has hydroxyl groups in its structure that are directly linked to its antioxidant activity, as described in the literature [33]. This suggests that in the presence of analog **D**, the formation of reactive oxygen species, responsible for oxidation of L-DOPA to dopaquinone was more difficult [3,18,19].

The lower tyrosinase inhibitory activity demonstrated by analog **E** compared to analog **D**, may be due to the insertion of the hydroxyl on the phenolic ring in the *ortho*-position. Molecules containing a *para*-hydroxyl (position 4) were more effective than molecules substituted in the *ortho*- or *meta*- (positions 1 and 2, respectively) [33,34,35]. Satooka and Kubo [28] described that the presence of a hydroxyl at position C4’ is essential for the inhibition of tyrosinase.

**Table 1:** Tyrosinase inhibitory activity of azaresveratrol analogs and kojic acid.

Analogs (n = 6)	Chemical Structure	IC 60 min (%)	IC 120 min (%)	IC_50_ (µg/mL)
**A**	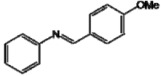	46.43 ^a^	38.81 ^f^	44.89 ^ij^
**B**	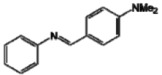	46.69 ^a^	44.6 ^fg^	72.58 ^ij^
**C**	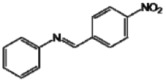	36.23 ^b^	31.20 ^f^	160.1 ^k^
**D**	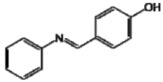	78.16 ^c^	71.97 ^h^	28.66 ^ijl^
**E**	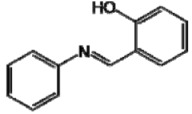	71.66 ^c^	68.49 ^h^	49.47 ^i^
**F**	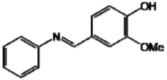	59.88 ^d^	51.59 ^gh^	147.96 ^k^
Kojic acid	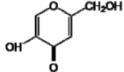	90.12 ^e^	75.92 ^h^	5.27 ^l^

Means followed by different letters differ by ANOVA followed by Tukey *post hoc* test (*p* < 0.05).

Regarding the analog **F**, its antioxidant potential assigned to a *para-*hydroxyl may have been diminished due to the methoxyl group inserted in the *meta*-position adjacent to the hydroxyl, and it increases the period of induction of oxidation compared to a non-methoxylated control [35,36].

However, Scotti *et al.* [37] have indicated that rings with *ortho*- and/or *para*-hydroxyl group substituents that also have other nitrogen or oxygen-containing substituents retain their antioxidant capacity due to resonance effects between electron pairs and a phenoxy radical, as seen in analog **A**.

The analog **C** presents a nitro group at position C4'. This compound may chelate metals and block the action of tyrosinase due to the unshared pair of electrons in its molecular structure that is able to complex with copper [38]. This happens because tyrosinase is a copper-protein enzymatic complex that requires copper ions to promote the redox reactions, essential in production of melanin [39]. The insertion of a disubstituted amine (analog **B**) on the parent molecule was not an useful modification for the tyrosinase inhibitory activity [20].

Phenolic compounds, such as the analogs tested, form relatively stable intermediates because of the resonance of the aromatic ring present in their structure [20]. This could explain the greater stability of the molecules proposed in this study compared to kojic acid.

**Figure 2:**
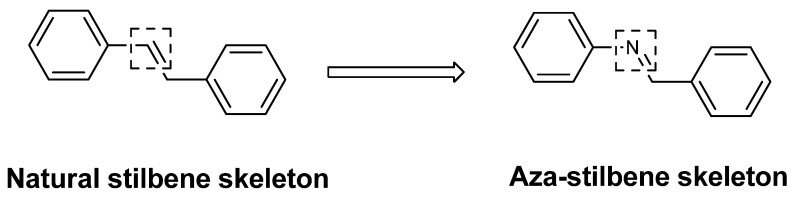
Comparison of thebasic structures ofnatural and aza stilbene skeleton.

Bernard and Berthon [13] determinated that the IC_50_ for resveratrol was 57.05 µg/mL. Therefore, the analogs **A**, **D** and **E** showed IC_50_ values lower than resveratrol, which demonstrated the great inhibitory potency of the analogs, superior to the natural compound. These three compounds, like resveratrol that has oxygen-containing groups in its structure, suggest that changes to the basic structure of resveratrol, where the CH grouping was replaced by a nitrogen atom, resulted in an increase in tyrosinase inhibitory effects (Figure 2).

Although only six analogs have been analyzed in this study, it is possible to observe that molecules with polar groups, such as hydroxyl, confer a higher tyrosinase inhibitory activity than analogs with fewer polar groups such as methoxyl and amine, highlighting the importance of a polar substituent on the molecule.

## 3. Experimental

### 3.1. Samples

**Scheme 1:**
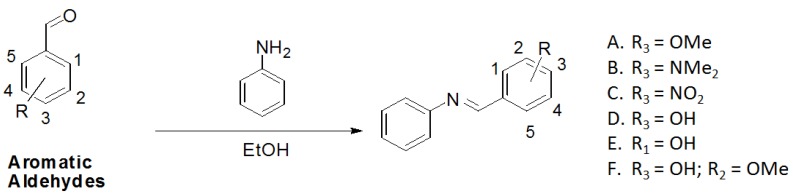
Synthetic pathway for aza-stilbene derivates.

Utilizing the classical method of imine formation, the six azaresveratrol derivates **A**–**F** were synthesized through condensation between aniline with a variety of aromatic aldehydes in ethanol [31,32] (Scheme 1). All compounds were characterized by ^1^H- and ^13^C-NMR, recorded on a BRUKER AVANCE DRX300, infrared (IR, BOMEM-FTIR MB-102) and melting point values (Table 2) which were in accord with literature data [21,40,41,42,43,44,45].

**Table 2:** Spectral data of aza-stilbene derivatives.

Compounds	δ C*H*=N	δ *C*=N	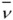 C=N	Melting Point (°C)	Yield (%)
**A**	8.51	159.8	1602	61.4–62.1	65.0
**B**	8.39	159.9	1600	96.8–97.3	72.0
**C**	8.80	158.8	1600	89.6–90.7	75.0
**D**	8.44	160.0	1602	89.2–90.7	74.0
**E**	8.96	163.5	1614	50.7–51.4	63.0
**F **	8.43	160.2	1622	53.1–54.2	63.0

* NMR experiments (ppm) were performed at 300 MHz for ^1^H and 75 MHz for ^13^C in dimethyl sulfoxide (DMSO-*d_6_*) and IR experiments (cm^−1^) were performed using KBr disks.

### 3.2. Preparation of Samples

The samples were dissolved in 25% aqueous dimethyl sulfoxide (DMSO) solution to obtain solutions with concentrations between 35–350 mg/mL, used in the assay of tyrosinase inhibition activity.

### 3.3. Test for Tyrosinase Inhibitory Activity

The ability to inhibit the activity of tyrosinase enzyme was evaluated using the enzymatic method described by Macrini *et* *al.* [46], with modifications. This method relies on the inhibition of tyrosinase in the presence of its substrate tyrosine, interrupting the synthesis of melanin.

#### 3.3.1. Tyrosinase Inhibition Qualitative Enzymatic Reaction Screening

Aliquots (10 µL) of a solution composed of 125 U/mL of mushroom tyrosinase (Sigma-Aldrich, St. Louis, MO, USA) were added to 96-well microplates, and then pH 6.8 phosphate buffer solution (70 µL) and the analogs (60 µL, 350 µg/mL, in 2.5% DMSO) were also added. For the positive control, kojic acid (60 µL, 17.5 µg/mL in 2.5% DMSO) was used instead of the analogs, and for the negative control 2.5% DMSO (60 µL) was added. To the resultant mixture, L-tyrosine (70 µL, Sigma-Aldrich) at a concentration of 0.3 mg/mL in distilled water was added (final volume in the wells = 210 µL). The absorbances of the microplate wells were read on a spectrophotometric microplate reader (SpectraCount, Packard, Meriden, CT, USA) at 510 nm (T_0_). Then, the microplates were incubated at 30 ± 1 °C for 60 minutes and the absorbances were registered again (T_1_). An additional incubation for 60 minutes at 30 ± 1 °C was done and after this period a new spectrophotometric reading was conducted (T_2_). The inhibitory percentage at the two times (T_1_ and T_2_) was obtained according to the formula to inhibitory activity percentage as follows:

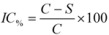
(1)
Where *IC_%_* = inhibitory activity; *C* = negative control absorbance; *S* = sample or positive control absorbance (absorbance at time T_1_ or T_2_ minus the absorbance at time T_0_).

#### 3.3.2. Tyrosinase Inhibition Quantitative Enzymatic Reaction Assay

For the samples that reached an IC greater than 50%, a quantitative assay was conducted. For this purpose, the above experimental protocol was followed, with modifications (a 500 U/mL tyrosinase solution was used instead of the 125 U/mL and the absorbance was measured every 10 min, for 1 h). The quantitative determination was obtained through an analytical curve and its respective linear equation. For this, the analogs were diluted in the microplate wells to five concentrations between 35 and 350 µg/mL with 25% DMSO, and the kojic acid was diluted to concentrations of 10, 5, 2.5, 1.25 and 0.625 µg/mL. Samples were assayed in triplicate. The curve showing tyrosinase inhibition activity percentages at each time point and the concentrations of the analogs/ positive control was plotted. The inhibitory activity at 50% (IC_50_, in µg/mL) was calculated using of the linear equation.

### 3.4. Statistical Analysis

We performed a descriptive statistical analysis and ANOVA followed by the Tukey *post hoc *test, with the Statistical Package for Social Sciences (SPSS) v.14.0 for Windows software, to compare the average values obtained between the resveratrol analogs and resveratrol analogs *versus* positive control (kojic acid) standard. The level of significance was 5%.

## 4. Conclusions

According to the data, *para*-hydroxylation was the molecular modification that gave the best evaluated tyrosinase inhibitory activity. However, this was lower than that of the reference standard tested. Resveratrol analogs may be important compounds to provide skin whitening and some analogs tested (**A**, **D** and **E**) showed lower IC_50_ values than resveratrol, the natural compound. Some of these aza-stilbenes were evaluated in recent studies for their antioxidant [21,32] and antituberculous effects [30] and together with the present results, indicate that these molecules may have pharmacological utility in a near future. Studies of the ratio between the *in vitro* behavior of the new analogs and their *in vivo* activity may contribute to the development of more effective skin whitening treatments.
